# The correlation between transient osteoporosis of the hip and pregnancy: A review

**DOI:** 10.1097/MD.0000000000035475

**Published:** 2023-10-13

**Authors:** Athanasios Galanis, Stefania Dimopoulou, Panagiotis Karampinas, Michail Vavourakis, Eftychios Papagrigorakis, Evangelos Sakellariou, Spyridon Karampitianis, Dimitrios Zachariou, Marianna Theodora, Panagiotis Antsaklis, George Daskalakis, John Vlamis

**Affiliations:** a 3rd Department of Orthopaedic Surgery, National & Kapodistrian University of Athens, KAT General Hospital, Athens, Greece; b 1st Department of Obstetrics and Gynecology, National & Kapodistrian University of Athens, Alexandra General Hospital, Athens, Greece.

**Keywords:** hip, osteoporosis, pregnancy, review, transient osteoporosis

## Abstract

Transient osteoporosis of the hip is indubitably a comparatively infrequent entity affecting both men and women worldwide. Its occurrence in the course of pregnancy, specifically in the third trimester, and lactation are of paramount concernment. The exact association between transient hip osteoporosis and pregnancy is precarious. Etiology and potential pathophysiological mechanisms behind this correlation are still to be utterly defined. Magnetic resonance imaging is highly regarded as the gold standard imaging method for assiduous assessment of this disorder. Physicians of copious medical specialties should practice scrupulous techniques for early and pertinent diagnosis when pregnant women are presented with persistent hip pain, as differential diagnosis with femoral head avascular necrosis can be exceedingly arduous. Treatment is predominantly conservative with protected weight-bearing and analgesic medication in the first line of management. In terms of prognosis, the disease ordinarily resolves spontaneously after a few months. Further research is required in order to elucidate the ambiguity surrounding the establishment of globally approved diagnosis and treatment guidelines for pregnancy-associated transient hip osteoporosis. This paper aims to accentuate the significance of this particular disorder by providing a succinct review of the existing literature, augmenting clinicians’ knowledge about the features of pregnancy-related transient proximal femur osteoporosis.

## 1. Introduction

Pregnancy-correlated transient osteoporosis of the hip (TOH) is an infrequent but also benign skeletal disorder, self-resolving in the vast majority of cases with a good prognosis, while only little is known regarding its etiology and overall incidence.^[1–3]^ TOH is common in both male and female middle-aged population, being more regularly reported in pregnant women during the third trimester and breastfeeding period as pregnancy is featured as the most common risk factor, whilst hip fractures scarcely occur.^[2–5]^ Hip is the most ordinarily affected site (76%), although knees, feet and ankles can be afflicted as well. Hip movement is usually limited by a rapid-onset hip pain, expanding to the groin and thigh, triggering weight-bearing arduousness.

The first 3 cases of TOH in pregnancy were reported by Curtiss and Kincaid in 1959, presenting patients with unilateral or bilateral hip pain or groin pain in the third trimester of pregnancy. Radiology evaluation demonstrated severe osteopenia of the femoral head, femoral neck and acetabulum, which exhibited rapid recovery several months postpartum, while similar cases were delineated at a subsequent time.^[6]^

Etiology and pathophysiology of pregnancy-associated TOH are still vague. Conventionally, osteoporosis is correlated with estrogen deficiency. Contrariwise, pregnancy is a state of excess estrogen production, while osteoporosis, which is chiefly unilateral, is presumably arising from impaired venous return and bone marrow edema.^[5,7,8]^ It is not uncommon for some patients to display past medical history of conditions linked to decreased bone mineral density (BMD) and bone mass. When these conditions are linked with the mechanical and metabolic stress during pregnancy and breastfeeding, a rise in bone turnover along with venous hypertension and/or microfractures can cause edema.^[5,7,8]^ A rare complication can be progression to avascular necrosis (AVN).^[8]^

Additionally, it has also been documented that women demonstrating no risk factors can potentially develop pregnancy-associated TOH. During lactation period, loss of bone mass can be inexorable, prompting an evanescent fracture-risk upsurge. Breastfeeding discontinuation induces bone mass recovery at a considerable extend, suggesting that halt of lactation may even have a protecting affection against the long-term evolution of osteoporosis or fragility fractures.^[7–9]^ It has not been determined whether osteoporosis medication approved in general population should be used during pregnancy, strikingly because there is lack of pertinent evidence and cause pregnancy-associated TOH is regarded as a benign and self-limiting condition.^[9]^

It is pivotal to emphasize that pregnancy-associated osteoporosis (PAO) is a distinct material malady, which might also transpire in physically-fit pregnant women in the third trimester or during the first months postnatal. Predominantly, fractures respond to the thoracolumbar spine and principally from T11 to L2, causing significant height loss.^[9]^ Several theories exist concerning PAO etiology and pathophysiology, but the exact cause is yet to be defined.^[9]^

In this paper, an overview of present-day knowledge surrounding the etiology, management and therapy of pregnancy-associated TOH is presented. Owing to the lack of consensus concerning classification, treatment and outcome scores, establishing apposite guidelines or algorithms is exceedingly strenuous. We endeavor to provide an up-to-date knowledge that might guide the obstetricians/gynecologists and orthopedic surgeons when dealing with pregnancy-associated TOH, bolstering their expertise among the variety of clinical presentations and the optimal combination of therapies.

## 2. Materials and methods

Literature search was conducted utilizing the MEDLINE/PubMed and Google Scholar databases for articles published from 1959 up to 2023. Keyword search terms were: “transient,” “osteoporosis,” “hip” and “pregnancy.” Language filters were activated for English. No restrictions were implemented in terms of the scientific articles’ publication date. Inclusion criteria were clinical studies, case series, reviews and papers reporting clinical cases. Exclusion criteria were papers presenting trials or cases about PAO and AVN in pregnancy. Articles in full text were scrutinized to retrieve additional relevant studies. Article selection was executed independently by 2 authors that employed the aforementioned inclusion criteria, whilst disagreements were elucidated with the contribution of a third author that made the decision. The collected data were entered into an Excel spreadsheet.

## 3. Results and discussion

**Figure 1.**
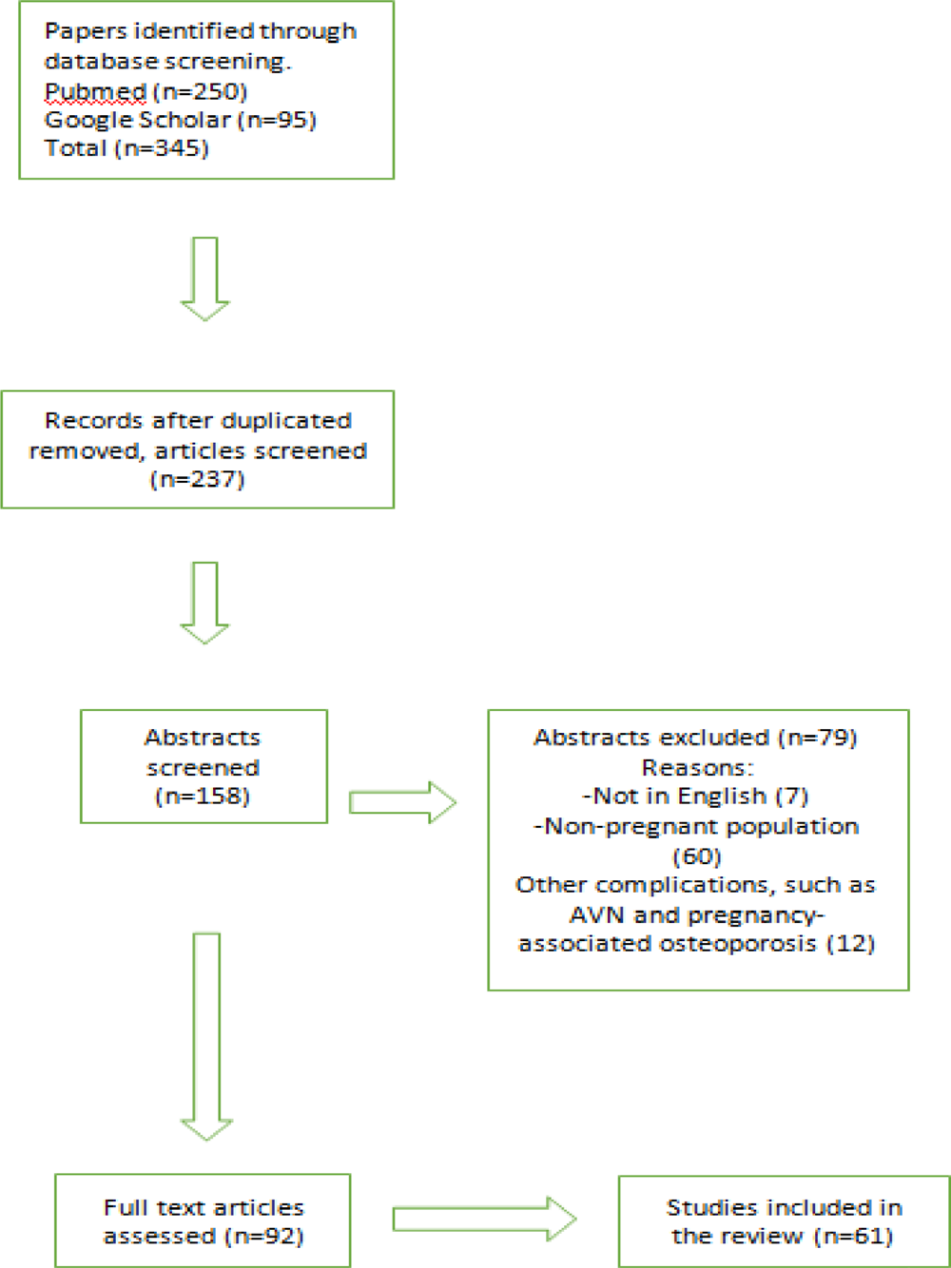
Study design.

A total of 345 papers were reviewed and 181 articles were selected for rigorous assessment. Following a full-text review, 11 additional articles were included from manual bibliographic search, perusing the pages of key journals and scanning reference lists of identified articles and documents. 61 papers were finally selected for citation (Fig. 1, Table 1).

**Table 1:** Main reviewed resources.

Authors (yr)	Article type	Country	Purpose of review	Type of source	Summary points
Møller U.K. et al (2012)	Cohort Study	Denmark	Amelioration of understanding of physiological changes in bone mass and body composition during pregnancy and postpartum	Medline/Pubmed	A decline in bone mineral density (BMD) occurs during pregnancy. During postpartum, BMD reduces further and is related to the length of breastfeeding period. Reversal of pregnancy-induced changes in body composition is also associated to breastfeeding status, as the decrease in fat mass postpartum is postponed in women who continue to breastfeed.
Mícheál Ó. Breasail et al (2020)	Cohort Study	UK	Quantifying the extent of pregnancy-induced alterations in compartmental BMD and bone mineral microarchitecture between the early second and the third trimester in UK women.	Medline/Pubmed	This study demonstrated for the first time within-pregnancy discrepancies in both BMD and bone microarchitecture. These changes underline the need for further research to determine the impact of reproduction on the maternal skeleton in the decade(s) preceding menopause as they may contribute considerably to the Ca requirements of the fetus.
Kovacs C.S. et al (2016)	Review	Canada	Review of current knowledge regarding maternal adaptations in mineral and skeletal homeostasis that transpire during pregnancy, lactation, and post-weaning recovery.	Medline/Pubmed	Pregnancy and lactation invoke novel regulatory systems in women to meet the challenges for increased delivery of minerals. In the long term, most studies delineate that parity and lactation pose no adverse risk of low bone mass or fractures, and in fact a number of studies have suggested that parity and lactation confer a protective effect. Lastly, these adaptations during pregnancy and lactation have important impact on preexisting disorders of bone and mineral metabolism.
Sowers M. et al (1993)	Cohort Study	USA	To test the apriori hypotheses that significant bone loss occurs in lactation of >5 mo duration and that bone mass returns to baseline levels when breast-feeding ceases.	Google Scholar	Extended lactation is correlated to bone loss. Notwithstanding, there is evidence of return to baseline BMD calculations at 12 mo after parturition. There is narrow evidence to justify any therapeutic intervention.
Holmberg-Marttila D. et al (2000)	Cohort Study	Finland	To examine physiologic and habitual factors, involving both lactation and amenorrhea, that would best explain changes in BMD during postpartum amenorrhea (PPA) and a subsequent follow-up period after resumed menstruation.	Medline/Pubmed	A systematic bone loss takes place during PPA, and after resumption of menstruation BMD recovers despite continued lactation. Nonetheless, the time of bony recovery back to postpregnancy levels appears to be modulated marginally by lactation habits.
Montella B.J. et al (1999)	Cohort Study	USA	To investigate the rare association of bone marrow edema syndromes of the femoral head and pregnancy	Google Scholar	Occasionally, pain in the hip that begins during pregnancy is caused by osteonecrosis of the femoral head. A high index of suspicion and use of magnetic resonance imaging may lead to an earlier diagnosis and a better prognosis in this population of women.
Hernigou et al (2018)	Case-crossover Study	France	To scrutinize the hypothesis that pregnancy is correlated with an enhanced risk of osteonecrosis and related conditions in healthy women.	Medline/Pubmed	For women without any risk factors or established preexisting conditions, a risk of hip osteonecrosis is present during the end of pregnancy and after delivery, and appears to decrease quickly. This may be helpful when evaluating symptoms concerning hip pain in pregnant or postpartum patients.
Quaresima P. et al (2021)	Review	Italy	It described the second reported Italian case of unilateral pregnancy related transient osteoporosis of the hip (PR-TOH) diagnosed by magnetic resonance imaging (MRI) and conducted a literature review	Medline/Pubmed	It accentuates the significance of correct diagnosis of PR-TOH in avoiding potential maternal complications such as fractures, and in reducing the risk of cesarean section and acquiring the best pregnancy outcome. An early diagnose made up by clinical and radiological (MRI) findings is of utmost importance
Hadji P. et al (2017)	Case control study	Germany	Assessment of risk factors for TOH in a homogenous population of women with TOH and an equal number of healthy matched controls. Also, identifying preventative charac- teristics in connection with this disease.	Medline/Pubmed	The etiology and underlying mechanisms of PR-TOH are still vague. This study supports the hypothesis that PR-TOH is multifactorial disease. Hereby, ther are significant associations with immobility, severe dental problems, and lack of exercise in childhood. Future studies on larger samples should be performed to further investigate risk factors and possible interventions in women with PR-TOH.
Cecile C.M. Röst et al (2004)	Cross-sectional study	Netherlands	The purpose of this study was to report the signs and symptoms of pregnant women with pain and dysfunction in the pelvic area.	Medline/Pubmed	Pregnant patients with pelvic pain exhibited a material level of complaints. The overall severity of complaints is not associated to previous peripartum pelvic pain or type of deliveries or to commonly used tests. Further study on the role of clinical examination, includ- ing passive flexion and internal rotation of the hip joints, was propounded
Sarah Steib-Furno et al (2007)	Prospective survey and Retrospective study	France	To prospectively evaluate the incidence of pregnancy-related hip diseases in an Obstetrics unit over a 2-yr period and to retrospectively review all gestational and postpartum hip disease cases recorded in a Rheumatology department over a 15-yr period	Medline/Pubmed	Hip diseases resulting in significant clinical impairment appear infrequent during pregnancy and early postpartum. Transient osteoporosis of the hip (TOH) was the most frequently encountered pregnancy-related hip disease. Contrariwise, osteonecrosis was rare. Stress fractures of the femoral head seem an important cause of pregnancy-related hip disease. Diagnosis of pregnancy-related covert fractures of the femoral head is cardinal because it might reveal an underlying bone disease.
Shlomi Toussia-Cohen et al (2023)	Retrospective case series study	Israel	To survey the natural course of TOH during pregnancy and postpartum in a large case series from a single tertiary center.	Medline/Pubmed	Transient hip osteoporosis is a self-limiting disease observed during pregnancy and postpartum. Opting for vaginal birth may be a fruitful option that does not expose pregnant women with TOH to pernicious outcomes compared with CD. Awareness of this potential diagnosis during pregnancy and postpartum is requisite. Further research is essential
K. Asadipooya et al (2017)	Review	USA	A literature review to retrieve articles cited on PubMed between 1959 and May 2014 concerning transient hip osteoporosis	Medline/Pubmed	MRI is the optimal method to diagnose TOH and to rule out traumatic injury, fracture, degenerative processes, inflammatory diseases and other conditions. Pregnant women are at risk of femoral neck fracture. However, most treatments are contraindicated. In pregnancy, calcitonin might shorten the duration of recovery which may prevent this complication. Yet, further research and randomized clinical trials are necessary
Konstantinos N. Malizos et al (2004)	Retrospective study	Greece	To describe the MRI findings, involving perfusion imaging, in a group of patients with a bone marrow edema (BME) of the hip and a final diagnosis of TOH.	Medline/Pubmed	TOH should always be considered in the differential diagnosis of a BME of the hip in young and middle-aged patients. Both physicians and patients should be aware of the possibility of acetabular involvement and of recurrence of this condition. Opportune differentiation of TOH from AVN will avoid unnecessary surgical intervention and facilitate appropriate management. These findings were in agreement with other papers that TOH is a distinct clinical entity, with a benign course.
L. Kibbi et al (2007)	Case report	USA	Report of 2 cases of transient osteoporosis of the hip and 1 case of transient osteoporosis of the knee, where the employment of oral bisphosphonates provided satisfactory outcomes	Medline/Pubmed	The 3 cases corroborated the data in the existing literature that bisphosphonates might be beneficial in diminishing the duration of transient osteoporosis. Well-controlled studies, however, are needed to confirm the findings.
Thurayya K. Arayssi et al (2003)	Case report and literarature review	Lebanon	Report of 2 patients with TOH during pregnancy that featured rapid resolution of their illness with the utilization of calcitonin and literature review focusing on TOH treatment	Medline/Pubmed	TOH is an under-recognized entity connected with pain and disability. The use of antiresorptive agents may be beneficial in dwindling the duration of the disease.
Aleksandra Truszczynska et al (2012)	Case report and literarature review	Poland	To present a case of transient peripartum femoral head osteoporosis that occurred twice at the same patient.	Medline/Pubmed	Transient hip osteoporosis is a clinical problem that has to be widely considered in cases of pregnant women presenting with spine and hip pain. Peripartum osteoporosis is regarded as a disease that often goes undiagnosed. It needs to be highlighted that self-limiting transient osteoporosis diagnosed at the right time and treated appropriately can be fully healed.
Charles Bircher et al (2011)	Case report	UK	To present a case of transient peripartum osteoporosis that resulted in bilateral fracture of the neck of the femur	Medline/Pubmed	The presented case underlined a rare complication that obstetricians should be aware of. When a woman complains of hip or pelvic pain, misdiagnosis of TOH can occur even with severe pain. It is exceedingly important to distinguish between hip fracture and other more benign causes of hip pain, because failing to diagnose these fractures early on may prompt displacement of the fracture, which has a potentially more severe orthopedic outcome.
Mary H.H. Ensom et al (1999)	Systematic review	Canada	To carry out a systematic review, with MEDLINE and Cochrane Library data base searches and bibliographic reviews, of English-language reports reporting therapy with low-molecular-weight heparin (LMWH) in pregnancy.	Medline/Pubmed	Although the risk of osteoporotic fractures correlated to unfractionated heparin is limited (≤2%), subclinical depletion of bone density can occur in 1-third of women receiving the agent long-term. It is contentious whether the reduced bone density connected with therapy is substantially greater than the impact of pregnancy alone on bone density. Also, it is debatable whether dosage and duration of heparin therapy correlate with the extent of osteopenia.
M. Backos et al (1999)	Prospective study	UK	Prospective study of the bone mineral density (BMD) alterations during pregnancy and the puerperium in 123 women with primary antiphospholipid syndrome treated with low-dose aspirin and subcutaneous low-dose heparin	Medline/Pubmed	Pregnant women demanding thromboprophylaxis can be encouraged that the loss in lumbar spine BMD related to low-dose long-term heparin treatment is similar to that which happens physiologically during pregnancy.

AVN = avascular necrosis.

### 3.1. Skeletal adaptations during pregnancy/lactation and pathophysiology

Pathophysiology of pregnancy-related TOH is equivocal, though several hypotheses have been postulated.

Affected regions are characterized by bone marrow edema and enhanced bone turnover.^[10]^ A combination of factors suggested to tally with this condition, are pregnancy-associated factors such as: immobilization, femoral venous stasis, obturator nerve pressure and hormonal variations of pregnancy, apart from traditional breastfeeding and osteoporosis risk factors involving: infective or inflammatory diseases, previous trauma, vascular lesions, neoplasia, alcoholic consumption, smoking, steroids, medications intake, hypothyroidism, low vitamin D or osteogenesis imperfecta.^[4,11]^

During pregnancy, several maternal adaptations occur for supplying the required calcium. The proportion of intestinal calcium absorption doubles, which is featured to be principally expedited by raised 1,25-dihydroxyvitamin D (1,25-OH D) levels generated by the maternal kidneys,^[11,12]^ albeit, other factors such as prolactin and placental lactogen may also contribute.^[11]^ Other alterations include parathyroid hormone (PTH) diminution and PTHrp increase precipitated by the placenta and bosoms, which are considered to conduce to the augmentation of 1,25-OH D by stimulating renal 1-alpha hydroxylase.^[12]^ Serum calcium dwindles in parallel with albumin, but levels of ionized calcium do not alter.^[12]^

Studies of BMD variations occurring in human pregnancy are broadly considered limited, contrasting BMD calculations conducted before pregnancy with those acquired postnatal. The biggest study examining BMD alterations in pregnancy was carried out by Moller et al in 2012.^[13]^ This study recorded 92 women who got pregnant, performing DEXA scans of lumbar spine, hip, and total body before pregnancy and then at 2 weeks, 4 months, and 9 months postnatal, respectively. Additionally, DEXA scans of forearm were conducted during each pregnancy trimester. The research involved a control group of 75 women who did not get pregnant for juxtaposition. 73 and 57 women in the corresponding groups, got through the first follow-up. BMD in the pregnant group was found to decline by a mean of 1.8 (±0.5%) in terms of the lumbar spine, 3.2 (±0.5%) regarding total hip and by 2.4 (±0.3%) concerning the total body, contrasting pre-pregnancy figures with those measured 2 weeks postnatal. Ultra-distal forearm BMD revealed a noteworthy, gradual abatement in pregnancy, and total forearm BMD was pronouncedly reduced by the 3rd trimester. Regulating for body weight/body composition alterations in pregnancy did not substantively modify these outcomes. Notwithstanding being statistically important, the documented BMD alterations in this research were minor.^[13]^ A conspicuous limitation of this paper was that by the time of 2-week postnatal follow-up visit, a crucial impact of early lactation cannot be precluded.^[12]^

Concerning bone structure, in a contemporary paper by Breasail et al, pQCT and HRpQCT were employed for collation of tibia and radius changes in pregnant women aged 30 to 45 with a control group of non-pregnant, non-breastfeeding females.^[14]^ In particular, 46 pregnant women and 37 non-pregnant controls were involved in that study. Reference scans at 14 to 16 weeks of pregnancy were contrasted to follow-up scans at a median of 35.8 weeks pregnancy. Larger pQCT figures depletion was observed in the pregnant group regarding total and trabecular vBMD, while greater decrease in total vBMD and cortical BMD was computed by HRpQCT. These alterations were predominately discernible in the tibia region, whilst concerning the radius area, solely the cortical bone-abundant proximal region demonstrated a discrepancy between the groups, with a cortical thickness reduction and enhancement in endosteal circumference detected in the pregnant group. The researchers conjectured that these outcomes might connote a differential pregnancy impact on bone architecture between weight-bearing and non-weight-bearing areas.^[14]^ Preponderantly, these data propound that albeit gestation does prompt some moderate affections on maternal skeleton regarding both BMD and bone structure, hormonal gestation adjustments secure that most fetal calcium necessities during pregnancy itself are attained via augmented calcium absorption from the mother sustenance.

Contrarily to gestation, during breastfeeding, maternal skeletal resorption is the principal adjustment guaranteeing an adequate calcium supply for milk-making. Skeletal calcium is produced by osteoclast-mediated bone resorption and osteolysis by osteocytes, incited by PTHrp from bosom tissue and little estradiol levels.^[12]^ Throughout breastfeeding, the kidneys do preserve calcium, however, intestinal calcium absorption returns to prior-pregnancy levels. Therefore, they are no more a salient factor in securing calcium requirements are fulfilled.^[12]^ Alongside that, circulating 1,25-OH D swiftly reverts to prior-pregnancy levels post childbirth.^[12]^ Maternal calcium input, either from diet or supplements, does not seem to amend intestinal calcium uptake rate or bone resorption, plainly resulting in upraised urinary calcium levels.^[12]^

Contrarily to pregnancy, throughout lactation, bone-turnover markers investigations reveal a noticeable rise in bone resorption markers compared with levels prior to/ during pregnancy.^[12]^ The majority of studies indicate that bone formation markers are raised as well, yet, to a smaller extent. This is indicative of uncoupling, prompting net resorption,^[12]^ a familiar resultant of estrogen insufficiency.^[15]^ BMD research exhibits weighty BMD reduction at all regions during 6 months of lactation. The biggest decrease (5%–10% averagely) is discovered in lumbar spine, with lower drop (up to 5%) at the hip/femur/distal radius areas, and only 0% to 2% lessening concerning total body calculations.^[12]^ This adduces that bone resorption throughout breastfeeding is more extensive at trabecular-rich areas, with cortical bone being less impacted. Nonetheless, there is an essential interindividual discrepancy concerning the amount of bone loss throughout breastfeeding, with a portion of women being deprived of up to 20% of their lumbar spine BMD, causing them to be osteoporotic.^[16]^ As anticipated, breastfeeding period is commensurate to the BMD abatement identified.^[17]^ Following the first 6 months, there are data suggesting that BMD does begin rising again in females carrying on lactation, presumably due to weaning that results in diminished feeding demand, nonetheless, at 12 months BMD is still found below reference levels.^[17]^ One paper denoted that BMD exhibits signs of recovery once menstruation recommences, even if breastfeeding carries on.^[18]^

Also, a mechanical factor has been implied for increasing the risk of TOH. Specifically, the left common iliac vein passes under the right common iliac artery, hence, the vein is more susceptible to compression. Taking into consideration the consequent excessive weight gain during pregnancy, this can trigger venous hypertension, intraosseous pressure, increased focal bone turnover and microfractures, notably concerning the left hip.^[19–22]^ Perusing literature, the left hip was most frequently involved, chiefly in the 3rd trimester or shortly postpartum, which is supportive of this mechanical theory.^[19,20,22,23]^ Though improbable, bone marrow edema may progress provoking vascular compression, leading to femoral head ischemic injury and AVN. AVN and hip osteonecrosis tend to respond in primigravida and in relatively elderly women.^[19,24]^

According to a literature review conducted by Quaresima P. et al,^[25]^ the majority of pregnancy-associated TOH cases have been reported without the presence of any risk factors. In a case control study, Peyman Hadji et al were the only ones to have investigated the potential definite pregnancy-related TOH risk factors. The outcomes pointed out that immobility, dental problems and lack of childhood exercise are eloquently correlated to the aforementioned disease.^[26]^

### 3.2. Clinical characteristics and etiology

Clinical presentation of TOH is customarily portrayed as an abrupt pain onset located in the groin region, anterior thigh and buttocks, which can involve one or both hips.^[9]^ Hip or pelvic pain throughout gestation is an accustomed complaint with an incidence rate ranging from 38% to 56%.^[27,28]^ Most of these complaints are benign, deriving from ligamentous strain at the pelvis and lumbar spine.^[29]^ Thus, vigilant physical examination is cardinal for distinguishing between hip and pelvic pathologies and can be eminently salutary, however, it can be also inaccurate and deceptive.^[30]^ In particular, TOH can be misdiagnosed as simple sciatica or sacroiliac strain.^[31]^ The most contemporary research concerning females diagnosed with TOH at a sole hospital was conducted by Toussia-Cohen S. et al.^[32]^ In this study, both obstetric and clinical features and findings of 34 pregnant females identified with TOH during gestation or postnatal period were reported and analyzed.^[32]^

The utmost findings of this study included: a comparatively increased maternal age (average age of mothers was 34.18 ± 4.75 years), the conspicuous preponderance of family history of non-PAO (29.4%) and escalated smoking proportions (47.1%), as well as women low prior-pregnancy body mass index (BMI) and at childbirth (22.03 and 27.60, correspondingly). Also, the proportion of in vitro fertilization (IVF) conception was relatively high (32.4%). Possible rationale behind the increased IVF conception rate might be the analogously increased median maternal age, along with the various medications employed in IVF treatments that have been formerly documented as TOH risk factors.^[9]^ In particular, 9 females (26.5%) were confirmed with bilateral TOH, a finding featuring material clinical significance, connoting that guidance involving weight-bearing abatement might be practicable in terms of both legs, hence, early imaging and diagnosis are vital. Clinical outcomes were not glaringly dissimilar between vaginal deliveries and c-section deliveries. What more, 2 women (13.3%) were reported with TOH in a sequential gestation.^[32]^

Regarding maternal age at the time of TOH diagnosis, in the review paper by Quaresima et al,^[25]^ average mothers age was 32.10 ± 5.50 years. Notwithstanding, Hadji et al^[26]^ reported 33 pregnant females identified with TOH in which the average age was found 35.20 ± 4.10 years.

In the study carried out by Toussia-Cohen S. et al, relatives medical record of pregnancy-unrelated osteoporosis was observed in 29.4% of cases, which was not documented in previous papers as a predisposing factor for TOH. It is vitally weighty to mention that the pathophysiological pathways of both entities are utterly dissimilar, and therefore the association betwixt the 2 is ambiguous. Moreover, at the same study, the percentage of smokers (47.1%) was notably large contrasted to studied smoking figures during gestation of just 6.7% in the United States in 2017.^[33]^ Consequently, smoking could be regarded a key predisposing factor for pregnancy-associated TOH as reported in preceding studies.^[34]^

A previous publication from Hadji P. et al revealed that women with TOH demonstrated a 2 kg bigger mean body weight contrasted to females without TOH,^[26]^ which is supportive of the mechanical theory. Nevertheless, these findings are conflicting with the study of Toussia-Cohen S. et al,^[32]^ which delineated that women BMIs, both prior-pregnancy and at childbirth (22.03 and 27.6, correspondingly), are comparatively small contrasted to average BMI figures in the population (24.7 and 29.7, correspondingly). Papers concerning non-transient osteoporosis have deduced that increased BMI ameliorates BMD, ergo reducing fracture danger, whilst small-scale BMI is a well-recorded risk factor for osteoporotic fractures.^[35]^

In terms of anticoagulants (mainly low molecular weight heparin [LMWH]) that are utilized as first-line agents for venous thromboembolism prevention, data regarding this treatment osteopenic effect are somewhat contradicting.^[35]^ Long-term LMWH prophylaxis in gestation for a minimum of 3 months has been correlated to bone loss and fractures,^[36–38]^ nevertheless, other studies indicated that the absolute fracture risk in this specific group was narrow (1%–2%).^[39]^ Additionally, BMD decreases of 2% to 4% prompted by the prophylactic doses of LMWH or unfractionated heparin were contiguous to the drop in bone mass that transpires customarily in gestation.^[40,41]^ In the study of Toussia-Cohen S. et al,^[30]^ antithrombotic prophylaxis owing to dwindled mobilization was employed in 29.4% of patients only for several months, with this limited use not expected to expose the women to an enhanced fracture danger.

Regarding delivery mode, a case-control study conducted by Hadji et al^[26]^ demonstrated that females with TOH featured increased rates of elective CD contrasted to females in good health. Also, Quaresima et al described TOH as a non-obstetric sign for cesarian section.^[25]^ In the study of Toussia-Cohen S. et al^[32]^ comparing delivery mode between women identified with TOH, only 5 women (31.2%) were admonished to proceed to elective cesarian delivery owing to TOH, this being the first report of such a comparison. This finding was ascribed to the cumulated familiarity with TOH patients, in addition to enhanced magnetic resonance imaging (MRI) scan availability that offers physicians the reassurance to permit pregnant females with TOH to attempt delivering vaginally.^[32]^

Concerning diagnosis timing, physicians ought to be tremendously aware of groin pain in the postnatal period, chiefly taking into account that medical monitoring at this period is patently limited than during gestation, considering carrying out an MRI scan in cases of acute persistent pain.

### 3.3. Differential diagnosis and imaging

Pregnancy-related TOH radiographic evaluation typically reveals the presence of osteopenia 4 to 6 weeks after the symptoms’ onset. However, MRI, which is indubitably a safe examination for pregnant women, is regarded as the optimum method for depicting bone marrow edema and abnormalities that can be descried as early as 48 hours after the emergence of symptoms.^[42,43]^ MRI findings indicative of TOH include intermediate signal sequences on T1-weighted image and high signal intensity on T2-weighted images.^[43]^ MRI is a safe examination for women

Furthermore, other MRI findings on contrast-enhanced imaging involve: hyperintensity, congruent enhancement pattern, no distinct borders with diffuse edema pattern, no focal defects, no subchondral alterations and delayed peak enhancement of the edematous marrow.^[42–45]^ TOH edema is regularly pinpointed at the femoral head and may extend to the femoral neck and intertrochanteric region, while being commonly accompanied by joint effusion.^[45,46]^

However, Malizos et al^[43]^ reported that, while there is there is no connection betwixt the extent of edema and the symptoms’ time-length, it appears that TOH with a spared subchondral zone resolves clinically more swiftly. It should be emphasized that patients varied in terms of the amount and extent of edema and subchondral changes, while the time interval between the onset of symptoms and MRI was disparate for each patient included in Malizos study. Contrariwise, a clinical study performed by Ergun et al illustrated that the clinical recovery length was commensurate with the extent of bone marrow edema and the size of subchondral fracture, if that was present.^[47]^ Additionally, Klontzas et al portrayed that the duration of symptoms is statistically associated with the extent of edema, but not with subchondral fracture(s).^[48]^

Differential diagnosis primarily includes AVN of the hip and less common septic arthritis and malignancy. In septic arthritis an inflammation of the joint and synovium is present. TOH and AVN of the hip are 2 separate clinical entities that exhibit copious distinguishing clinical and radiographic features.^[6]^ AVN MRI findings may reveal a distinct local lesion of the femoral head, subchondral radiolucency (crescent sign), single line sign with edema on T1-weighted image and double line sign with edema on T2-weighted image.^[46]^

According to a review by Quaresima et al, while X-rays are broadly the most utilized radiological technique after delivery, MRI has steadily become the gold standard for pregnancy-associated TOH diagnosis through years.^[25]^ Regarding X-ray findings, during the initial 3 to 6 weeks from the onset of symptoms they are usually absent, and when radiographic findings are evident, they include diffuse femoral head osteopenia or periarticular demineralization, yet, the femoral head ordinarily remains intact.^[43,46]^ What more, the lack of subchondral changes is a fundamental indicator of TOH.^[49]^ TOH blood flow and capillary permeability upsurge triggers a rise in radionuclide uptake, thus, a positive bone scan may be detected in all 3 phases and can carry on for weeks after clinical melioration.^[43,50,51]^ Whilst regional migratory osteoporosis is contiguous with TOH in that it is also ordinarily transient; it is characterized by its asymmetric involvement and movement from proximal to distal, advancing unilaterally from the hip region to knee and ankle.^[52]^ Taking into account that regional migratory osteoporosis is sporadically connected with BMD alterations, serial bone density calculations may denote the evolution of both bone loss and succeeding recovery of impacted regions.^[53]^

### 3.4. Prognosis and treatment

Recommended TOH treatment approaches during pregnancy include conservative options such as: non-weight-bearing, wheelchair employment, utilization of crutches for partial weight-bearing, progressive physiotherapy for preventing contractures of the involved hip and mild analgesics like paracetamol and nonsteroidal anti-inflammatory drugs. The objective of these treatments is preclusion of microfractures and pain alleviation, however, the natural course of the disease is not modified.^[51]^

Oral and intravenous bisphosphonates along with other antiresorptive agents are not suggested in pregnant and lactating women.^[54,55]^ Bisphosphonates exert influence on fetal skeletal development, causing preterm delivery, fetal growth restriction, neonate transient hypocalcemia and spontaneous abortion. Nonetheless, calcitonin does not cross the placenta and appears to be innocuous during pregnancy, having been shown to diminish symptoms’ duration.^[54,55]^ Additionally, the employment of calcitonin during pregnancy might confine TOH duration and recovery time.^[56]^ Treatment with calcium and vitamin D_3_ supplements can apparently be encouraged, since no pregnancy-related contraindications have been reported employing these drugs, with their use being advocated notably in the first phases of the disease.^[54–56]^

In cases where pregnant TOH patients are not aptly treated, or if TOH does not reverse, subsequent microfractures might bewilder the diagnosis with AVN. The evolving bone injury in conjunction with further bone edema increase might induce necrosis, bone collapse, articular distortion, and proximal femoral fracture.^[5]^ Debatably, TOH might be regarded as an early-stage precursor of AVN.^[5,8]^ Notwithstanding, while TOH generally resolves without sequelae, AVN is customarily an irrevocable and progressive disease, deriving from persistent femoral head blood supply interruption, occasionally provoking permanent joint failure.^[8]^ Total hip arthroplasty or internal fixation is the treatment of choice regarding displaced fractures caused by TOH, whilst internal fixation is opted for non-dislocated fractures and conservative management is proposed for subcapital fractures.^[57,58]^ A review study by Vergara-Ferrer et al^[57]^ examined different therapeutic options (closed reduction internal fixation or open reduction internal fixation with cannulated screws; with or without bone graft utilization and partial or total hip arthroplasty) during third-trimester pregnancy. Eventually, all except for one case healed with no subsequent necrosis, irrespective of the time elapsed since the fracture was discerned.^[57]^

The cohort study by Toussia-Cohen S. et al^[32]^ inquired into the results of LMWH usage for a limited period (3–6 months), inferring that LMWH, as used in the research, is not expected to expose pregnant patients to an increased fracture danger. Nonetheless, if LMWH treatment is projected for an expanded period, advantages and disadvantages should be gingerly assessed before treatment commencement.^[32]^

In general, TOH symptoms typically resolve within the first 2 months postnatal. In the majority of cases, MRI findings return to normal over a 3 to 6 month period after delivery.^[57,58]^ However, there have been documented cases resolving only after 6 years from diagnosis,^[58]^ while recurrence in subsequent pregnancies has not been described.^[26]^

No paper has highlighted TOH impact during pregnancy regarding the mode of delivery, although an elective Caesarean section has been widely featured to protect women from birth-related injuries.^[59]^ In the vast majority of pregnancy-associated TOH cases, the potential occurrence of severe hip pain and joint functional limitation has rendered this condition a non-obstetric indication for elective Caesarean section.^[59]^ According to the literature review conducted by Quaresima et al, more than 70% of Caesarean delivery has been executed with regards to the consequential hip functional limitation.^[25]^ It is remarkable that Caesarean section rates at this level are well above the World Health Organization recommended ones.^[60]^ In the most recent cohort study carried out by Toussia-Cohen et al, where clinicians with augmented experience with TOH patients were included, only 5 women (31.2%) delivered by elective Caesarean section due to TOH, while all women were given the option of vaginal delivery.^[32]^

## 4. Conclusion

TOH in pregnancy and lactation is preponderantly a self-limiting disorder, usually resolving 6 to 8 months postpartum. Etiology and pathophysiological mechanisms have not been consummately determined. In general, the disease is chiefly unilateral, presumably triggered by impaired venous return with bone marrow edema.

Opportune diagnosis is pivotal for optimal patient management, avoiding potentially debilitating effects resulting from other diseases of similar presentation, such as AVN of the femoral head. MRI is irrefutably the most apposite imaging examination and clinicians should demonstrate a low threshold for ordering an MRI scan when dealing with pregnant women with persistent hip pain.

Treatment is ordinarily conservative, including analgesics and protected weight-bearing for prevention of pathological fractures, commonly up to a few weeks postpartum. Bisphosphonates are not recommended during pregnancy but calcitonin might curtail recovery time duration. In cases of femoral neck fractures, surgical treatment can be a vital option, whilst conservative management is customarily proposed in terms of subcapital fractures.

Further research is requisite since there are no globally established strategies regarding consummate follow-up, therapy and clinical management of pregnancies complicated by TOH.

## Author contributions

**Conceptualization:** Stefania Dimopoulou.

**Data curation:** Dimitrios Zachariou, Marianna Theodora.

**Investigation:** Evangelos Sakellariou, Spyridon Karampitianis.

**Resources:** Panagiotis Antsaklis, Georgios Daskalakis.

**Supervision:** John Vlamis.

**Writing – original draft:** Athanasios Galanis, Panagiotis Karampinas.

**Writing – review & editing:** Michail Vavourakis, Eftychios Papagrigorakis.
